# Clinical Outcomes of Biliary Drainage in Patients with Malignant Biliary Obstruction Caused by Colorectal Cancer Metastases

**DOI:** 10.1007/s12029-022-00834-y

**Published:** 2022-05-24

**Authors:** Janine B. Kastelijn, Leon M. G. Moons, Jakob W. Kist, Jip F. Prince, Maarten S. van Leeuwen, Miriam Koopman, Frank P. Vleggaar

**Affiliations:** 1 Department of Gastroenterology and Hepatology, University Medical Center Utrecht, Heidelberglaan 100, 3584 CX Utrecht, The Netherlands; 2 Department of Radiology, University Medical Center Utrecht, Heidelberglaan 100, 3584 CX Utrecht, The Netherlands; 3 Department of Medical Oncology, University Medical Center Utrecht, Utrecht University, Heidelberglaan 100, 3584 CX Utrecht, The Netherlands

**Keywords:** Biliary drainage, Colorectal cancer, Liver metastases, Obstructive jaundice, Gastrointestinal endoscopy, Neoplasm metastasis

## Abstract

**Background and aim:**

Malignant biliary obstruction is an ominous complication of metastatic colorectal cancer (mCRC) that is challenging to solve. Biliary drainage can be performed to relieve symptoms of jaundice, treat cholangitis, or enable palliative systemic therapy. The aim of this study is to evaluate clinical outcomes of biliary drainage of malignant biliary obstruction in mCRC patients.

**Methods:**

Consecutive patients with malignant biliary obstruction due to mCRC who underwent endoscopic retrograde cholangiopancreatography or percutaneous transhepatic cholangiography were included. Patient, disease, and procedural characteristics and outcomes were retrospectively collected from electronic medical records. Radiological data were prospectively reassessed. Main outcome was functional success, i.e. achievement of the intended goal of biliary drainage. Prognostic factors for functional success and survival were assessed.

**Results:**

Thirty-seven patients were included. Functional success was achieved in 18 (50%) patients. Seventeen (46%) patients experienced adverse events (suspected to be) related to the procedure. Median overall survival after biliary drainage was 61 days (IQR 31–113). No prognostic factors of functional success were identified. Performance status, presence of the primary tumor, ascites, ≥ 5 intrahepatic metastases, estimated hepatic invasion of > 50% and above-median levels of bilirubin and lactate dehydrogenase were significantly associated with poorer survival. Improved survival was seen in patients with technical, functional, or biochemical success, and with subsequent oncologic treatment.

**Conclusions:**

Functional successful biliary drainage was achieved in half of the patients. Adverse events also occurred in nearly half of the patients. We observed a significantly longer survival in whom biliary drainage allowed palliative oncologic therapy.

## Introduction

Colorectal cancer (CRC) is the third most common malignancy globally [1]. Population-based series showed that 25–30% of CRC patients eventually develop liver metastases [2, 3]. Malignant biliary obstruction was reported in 10% of patients with known metastatic CRC (mCRC) and is usually an ominous finding [4]. It can be caused by intrahepatic metastases replacing normal liver parenchyma and obstructing bile ducts, or by extrahepatic lymph nodes or peritoneal metastases located along the extrahepatic bile duct or at the liver hilum causing mechanical obstruction.

Biliary drainage by endoscopic retrograde cholangiopancreatography (ERCP) or percutaneous transhepatic cholangiography (PTC) is commonly performed in palliative settings [5]. It aims to relieve symptoms of obstructive jaundice, treat complications such as cholangitis, or lower the hyperbilirubinemia to allow administration of palliative oncologic therapy. With supportive care alone, median survival after the onset of jaundice is around 1 month [4, 6]. When biliary drainage allows further oncologic therapy, survival up to 1 year has been reported [4, 7–9]. Clinically effective biliary drainage, however, can be difficult to achieve [4, 7–11]. In addition, every drainage attempt carries the risk of adverse events, such as cholangitis and pancreatitis, which may negatively impact survival and quality of patients’ last phase of life. Radiographical imaging of the biliary tract is usually performed before drainage, to locate the stricture and decide on the best therapeutic strategy and drainage options.

Data on short- and long-term clinical outcomes, the efficacy of biliary drainage in mCRC patients and their radiologic characteristics are scarce. Yet these data can guide and improve patient selection. We therefore aim to describe clinical outcomes of biliary drainage and to identify (radiologic) prognostic factors associated with functional success and survival, in a cohort of mCRC patients with malignant biliary obstruction.

## Patients and Methods

All patients with mCRC who underwent ERCP or PTC for biliary drainage of malignant biliary obstruction at our institution (University Medical Center Utrecht (UMCU), Utrecht, The Netherlands) between January 1, 2010 and December 31, 2018 were retrospectively identified and assessed for eligibility. Our study was approved by the Institutional Medical Research Ethics Committee of the UMCU (MREC number 19/698) and was performed in compliance with the General Data Protection Regulation (GDPR). This study adhered to the Strengthening the Reporting of Observational Studies in Epidemiology (STROBE) Guidelines [12].

### Inclusion Criteria

Patients with (1) mCRC; presenting with (2) the first episode of malignant biliary obstruction for which palliative biliary drainage by ERCP or PTC was performed; and in whom (3) malignant biliary obstruction was secondary to either intrahepatic metastases or extrahepatic lymph node of peritoneal metastasis from mCRC, were included. Biliary obstruction was confirmed by a dilated biliary system radiographically on either abdominal ultrasound (US), computed tomography (CT), and/or magnetic resonance imaging/cholangiopancreaticography (MRI/MRCP). Patients with other causes of jaundice (e.g., benign strictures, cholelithiasis, cirrhosis, or Gilbert’s syndrome) or patients in whom the first attempt of biliary drainage was performed in another hospital were excluded.

### Data-collection

The following patient and disease characteristics were retrospectively collected from electronic medical records: age, sex, World Health Organization (WHO) performance status, presence of the primary tumor, date of initial CRC diagnosis, date of hepatic metastases, date of obstructive jaundice, previous oncologic treatment (hepatic surgery, lines of chemotherapy), presence of fever, ascites, peritoneal metastases, and values of bilirubin and lactate dehydrogenase (LDH). The following procedural characteristics were retrospectively collected: date, type, and indication of biliary drainage, number of procedures performed until technical and functional success was achieved or until no further attempts were undertaken, type and number of stents that were used to reach the technical outcome, the presence of a permanent external drain to facilitate biliary drainage, administration of prophylactic antibiotics and rectal diclofenac. Uncertainties about eligibility and outcome adjudication were discussed with a senior gastroenterologist (FV).

All patients underwent radiological evaluation prior to biliary drainage, either by abdominal US, CT, and/or MRI/MRCP. All available data within 3 months prior to the intervention were assessed by two radiologists in training (JWK and JP, both with over 5 years of training). Scoring was performed independently and blinded to outcomes. Discrepancies were resolved in consensus meetings. The following radiological data were prospectively obtained and reassessed: the number (≤ 4, 5–9, 10–14 or ≥ 15) and size (≤ 5 or > 5 cm) of intrahepatic metastases; level of biliary obstruction (defined as ‘[peri]hilar’ when the hilum was involved, ‘suprahilar’ for main ducts or segmental ducts, and ‘infrahilar’ for intrapancreatic of hepatoduodenal ligament obstructions); and the location of the cause of obstruction (intrahepatic or extrahepatic).

### Procedural Techniques

All peri-procedural care was performed in accordance with local practice and guidelines at the time of intervention, and at the discretion of the endoscopist or radiologist.

### Outcome Measures

Our main outcome was functional success, defined as achievement of the intended goal of biliary drainage (allowing further oncologic treatment [chemotherapy, phase 1 studies, immunotherapy or radio-embolization], resolving cholangitis, or symptomatic relief), depending on the intention of biliary drainage.

Other outcomes of interest were technical success, biochemical success, adverse events, stent failure, and overall survival. Technical success was defined as successful drainage of the intended biliary stricture(s). Partial technical success was defined as successful drainage of some, but not all intended strictures. Post-procedural bilirubin was defined as the lowest post-procedural bilirubin measured within 3 months after the procedure. Biochemical success was defined as reduction of the bilirubin level to twice the normal value or less (≤ 42 umol/L) within 3 months after the procedure. Adverse events were defined as any potentially procedure-related adverse event occurring during or after biliary drainage, registered until death. Adverse events were defined and graded according to the American Society for Gastrointestinal Endoscopy (ASGE) lexicon, where applicable [13]. Stent failure was defined as recurrence of obstructive jaundice due to stent dysfunction, after initial clinical or biochemical success was achieved, requiring endoscopic or percutaneous reintervention. Overall survival was defined as time between the first biliary drainage and death. Clinical data were collected until death or last contact as reported in the electronic medical records on July 1, 2020. We considered patients free of stent failure if no symptoms of recurrent obstructive jaundice were recorded at last contact (physical or by telephone) or before death.

### Statistical Analysis

Data were presented as means and standard deviations (± SD) for continuous variables with normal distribution and medians with interquartile range (IQR) for continuous variables with a skewed distribution. Categorical variables were presented as absolute numbers and percentages. Univariable analyses to determine risk factors for functional success and survival were performed with logistic regression and Cox proportional hazard regression, respectively. The association between functional success and survival was adjusted for WHO performance status. Candidate prognostic factors were selected based on expert opinion and on the results of previous literature [7, 10, 11]. Continuous laboratory values were dichotomized based on their median level. Kaplan–Meier survival curves were created to assess whether functional success improved survival and were compared using the logrank test. This was done for the total cohort and for the intended goals ‘further oncologic therapy’ and ‘symptom relief’, separately. Results were considered statistically significant if the *p*-value was < 0.05. Subgroup analyses were not performed due to the small sample size. Statistical analyses were performed with STATA version 15.1.

## Results

Thirty-seven consecutive patients were included. Two patients were alive at the time of analysis in July 2020. Median duration of follow-up was 63 (IQR 33–159) days.

### Baseline Characteristics

Mean age was 62 years (± 9.4 years) and 70% was male. Twenty (54%) patients had been treated with more than one line of systemic therapy. Baseline characteristics are provided in Table 1. Twenty-one (57%) patients had synchronous liver or lymph node metastases at time of CRC diagnosis. In 12 (32%) patients, metastases were diagnosed at a median of 563 days (IQR 190–898) after CRC was diagnosed. Obstructive jaundice occurred at a median of 685 days (IQR 258–947) after diagnosis of liver or lymph node metastases. Of the remaining 4 (11%) patients, the exact dates of diagnosis of either CRC (n = 2) and/or the occurrence of liver or lymph node metastases (n = 2) were missing.

**Table 1:** Patient characteristics (n = 37)

Age in years, mean ± SD	62 ± 9.4
Sex, n male (%)	26 (70)
WHO performance status, n (%)	
0	4 (11)
1	22 (59)
2	6 (16)
3	5 (14)
Primary tumor removed, n (%)	29 (78)
Previous hemi-hepatectomy, n (%)	
Left	2 (5)
Right	7 (19)
(Partial) segment resection	6 (16)
No	22 (59)
Lines of chemotherapy, n (%)	
Chemo naïve	4 (11)
First line	13 (35)
Second line	9 (24)
Third line	7 (19)
Fourth line or more	4 (11)
Fever before drainage, n (%)	2 (5)
Ascites, n (%)	13 (35)
Peritoneal carcinomatosis, n (%)	9 (24)

### Disease and Procedural Characteristics

Laboratory and radiological characteristics are shown in Table 2. Biliary obstruction was caused by intrahepatic obstructions in 22 (59%) patients and by extrahepatic obstructions in 13 (35%) patients. Of the latter, 9 (69%) patients also had intrahepatic metastases.

**Table 2:** Disease characteristics

Preprocedural laboratory measures, median (IQR)	
Bilirubin (umol/L)^a^	135 (85–219)
LDH (U/L)^b^	365 (269–584)
Radiological modality used to extract data, n (%)^c^	
US	1 (3)
CT	22 (61)
US and CT	8 (22)
MRI/MRCP	5 (14)
Intrahepatic metastases, n (%)	
Yes	28 (78)
Total number of intrahepatic metastases	
1–4	14 (50)
5–9	3 (11)
10–14	1 (4)
15 or more	10 (36)
Maximum diameter of intrahepatic metastases	
≤ 5 cm	7 (25)
> 5 cm	21 (75)
Estimated hepatic invasion rate	
≤ 50%	25 (89)
> 50%	3 (11)
No	6 (17)
Not possible to determine radiographically	2 (6)
Cause of obstruction, n (%)	
Extrahepatic	13 (35)
Intrahepatic	22 (59)
Not possible to determine radiographically	2 (5)
Level of obstruction, n (%)^a^	
Suprahilar	5 (14)
(Peri)hilar	18 (50)
Infrahilar	10 (28)
Not possible to determine radiographically	3 (8)

^a^Missing value in one patient

^b^Missing values in four patients

^c^In 1 patient, radiological imaging was performed > 3 months before ERCP and was not used

Procedural characteristics are shown in Table 3. The indication for biliary drainage was allowing further oncologic therapy in 26 (70%) patients (chemotherapy in 16 patients, panitumumab in 2 patients, phase 1 study in 5 patients, radio-embolization in 2 patients, and local treatment with irreversible electroporation in 1 patient); lowering bilirubin to relieve symptoms in 9 (24%) patients; and to treat cholangitis in 2 (5%) patients. Thirty (81%) patients underwent one procedure (29 ERCP and one PTC) and 7 (19%) patients underwent two procedures (two ERCPs in 2 patients; ERCP + PTC in 5 patients, of which 2 followed by rendez-vous) until technical success was (partially) achieved, or until technical success was not achieved but further attempts were abandoned. Thirty (81%) patients received one stent and 6 (16%) patients received two stents (of whom 4 received two plastic stents, 1 received two metal stents, and 1 received a metal stent combined with a drain). All 6 patients with two stents had a perihilar biliary obstruction. The total number of procedures performed until functional success was achieved or after which no further attempts were undertaken ranged between 1 and 9.

**Table 3:** Procedural characteristics

Indication for biliary drainage, n (%)			
Further oncologic therapy	26 (70)		
Relief of symptoms	9 (24)		
Cholangitis	2 (5)		
Type of initial biliary drainage, n (%)			
ERCP	36 (97)		
PTC	1 (3)		
Number of procedures to reach technical outcome, n (%)	*Success*	*Partial*	*Failure*
1 procedure^a^	24 (65)	5 (14)	1 (3)
2 procedures^b^	7 (19)	–	–
Type of stent used to reach technical outcome, n (%)			
Plastic	16 (43)	4 (11)	0 (-)
Metal	9 (24)	1 (3)	0 (-)
Drain	5 (14)	0 (-)	0 (-)
Combination^c^	1 (3)	0 (-)	0 (-)
No stent	0 (-)	0 (-)	1 (3)
Total number of procedures until functional outcome, n (%)	*Success*	*Failure*	
1 procedure	10 (27)	12 (32)	
2 procedures	3 (8)	2 (5)	
3 procedures	3 (8)	-	
≥ 4 procedures	2 (5)	4 (11)	
Permanent external drain, n (%)	7 (19)		
Procedural antibiotics, n (%)	26 (70)		
Prophylactic diclofenac suppository, n (%)	18 (49)		

^a^ERCP in 29 patients and PTC in 1 patient

^b^ERCP-ERCP in two patients and ERCP-PTC in five patients

^c^Combination of a metal stent intrahepatically to the right and a PTC-drain intrahepatically to the left as this segment was opacified but not reached endoscopically

### Clinical Outcomes

Clinical outcomes are provided in Table 4.

**Table 4:** Clinical outcomes

Technical success, n (%)	
Yes	31 (84)
Partial	5 (14)
No	1 (3)
Functional success, n (%)^a^	18 (50)
Post-procedural bilirubin, median (IQR)^b^	70 (29–182)
Biochemical success, n (%)^c^	15 (44)
Subsequent treatment, n (%)	11 (30)
Adverse events, n (%)	17 (46)
Stent failure requiring reintervention, n (%)	11 (30)
Overall survival after biliary drainage in days, median (IQR)	61 (31–113)
30-day mortality, n (%)	8 (22)
Days of follow-up, median (IQR)	63 (33–159)

^a^Missing value in one patient

^b^Missing values in four patients

^c^Missing values in three patients

#### Technical and Biochemical Success

**Fig. 1:**
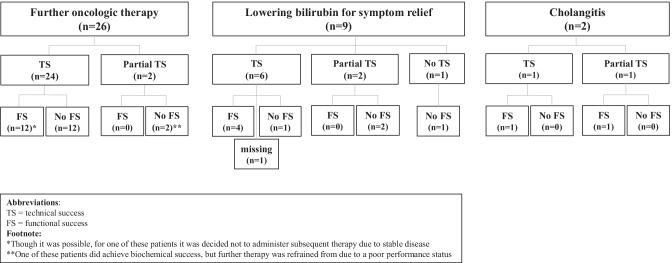
Flow chart detailing the outcomes technical and functional success after biliary drainage for each indication: further oncologic therapy, lowering bilirubin for symptom relief and cholangitis

Biliary drainage was technically successful in 31 (84%) patients and partially successful in 5 (14%) patients. Of 5 patients with partial technical success, 4 had a perihilar and 1 a suprahilar stricture. Four (80%) of these patients did not reach functional success. Technical success was not achieved in one (3%) patient in whom duodenal obstruction could not be passed with the endoscope. Additional treatments, such as a PTC-drain, were considered too burdensome for this patient. Technical outcomes of biliary drainage by each indication are shown in Fig. 1.

Of all the patients, 15 (44%) patients reached biochemical success. This was in 2 (100%) patients who underwent biliary drainage to treat cholangitis, none of the patients who were treated to relieve symptoms and in 13 (50%) patients who were treated to allow further oncologic therapy.

#### Functional Success

Functional success was achieved in 18 (50%) patients. Functional outcomes of biliary drainage by each indication are shown in Fig. 1.

Of the 26 patients in whom biliary drainage was performed to allow further oncologic treatment, functional success was achieved in 12 (46%) patients. Of these 12 patients, two had not been previously treated with systemic therapy, seven had been treated with first-line therapy and three with second-line therapy. In one patient, subsequent therapy administration was deliberately refrained from, after adequate treatment with stenting alone and limited tumor volume. Functional success was not achieved in 14 (54%) patients. In 12 of these patients, the bilirubin level remained too high to allow further oncologic treatment, often accompanied with clinical deterioration. One patient did not receive further treatment due to a poor performance status, despite normalization of bilirubin levels. One patient died as a result of a non-procedure–related adverse event before systemic therapy could be considered.

Of the 9 patients in whom biliary drainage was performed for symptom relief, 4 (50%) patients achieved functional success and experienced improvement of symptoms. Symptoms did not improve in 4 (50%) patients. One patient was lost to follow-up.

Both patients undergoing biliary drainage for cholangitis were functionally successful and recovered from cholangitis. One patient continued best supportive care treatment and the other patient developed post-ERCP pancreatitis and rapid progressive disease with hyperbilirubinemia and no further oncologic treatment options.

#### Adverse Events and Reinterventions

Seventeen (46%) patients experienced one or more adverse events after biliary drainage that were (suspected to be) related to the procedure. Eleven (30%) patients had one or more episodes of cholangitis, of which five due to stent failure after functional success was initially achieved. All were of moderate severity because it required repeat endoscopy or prolonged hospital admission for 4–10 days. Drain dislocation requiring reintervention (moderate severity) occurred in 4 (11%) patients who underwent biliary drainage (57% of the patients with a permanent drain for biliary drainage). Post-ERCP pancreatitis was reported in 4 (11%) patients: 1 of mild and 3 of moderate severity. One (3%) patient presented with fever without clear focus, requiring a prolonged length of hospital stay to administer antibiotics (mild severity).

Stent failure after functional success, causing cholestasis or cholangitis, occurred in 11 (30%) patients, with a median number of 3 (IQR 1–8) reinterventions. Time between the first ERCP and the moment of reintervention was a median of 99 days (IQR 48–210).

#### Survival

**Fig. 2:**
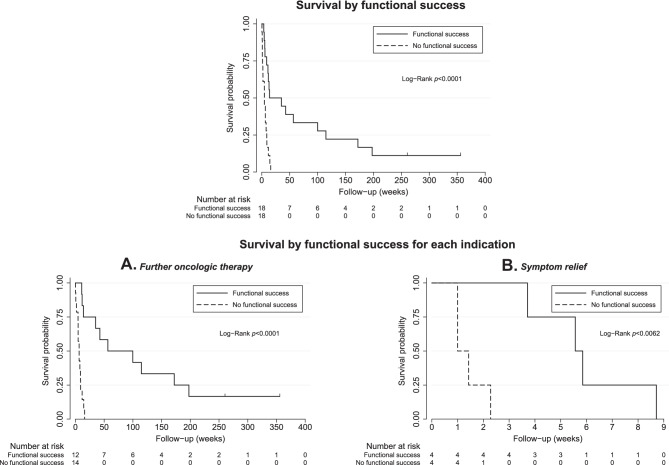
Survival by functional success in all patients and stratified by indication of biliary drainage, such as further oncologic therapy (**A**) or symptom relief (**B**)

Median overall survival after biliary drainage was 61 days (IQR 31–113); 95 days (IQR 51–548) in patients with and 34 days (IQR 10–63) in patients without functional success, *p* < 0.001 (Fig. 2).

Median survival was 81 days (IQR 40–273) in patients who underwent biliary drainage to allow further oncologic therapy. In this subgroup, survival was 348 days (IQR 98–804) in patients with and 46 days (IQR 33–65) in patients without functional success, *p* < 0.0001 (Fig. 2A).

In patients in whom biliary drainage was performed to relieve symptoms, median survival was 26 days (IQR 10–41); 40 days (IQR 33–51) in patients with and 9 days (IQR 7–13) in patients without functional success, *p* = 0.0062 (Fig. 2B).

Survival in the two patients treated for cholangitis was 32 and 92 days.

#### Prognostic Factors

For functional success, no significantly associated prognostic factors were identified in univariable analysis. For survival, univariable analysis showed that WHO 2–3 vs. WHO 0–1, presence of ascites, ≥ 5 intrahepatic metastases, and an overall estimated hepatic invasion of > 50% were associated with poorer survival. Survival was longer in patients in whom the primary tumor had been removed. In addition, values of bilirubin and LDH before drainage that were above the median value were associated with poorer survival. Survival was improved after technical, functional, and biochemical success, and after administration of subsequent treatment. After adjusting for WHO performance status, functional success remained significantly associated with improved survival (Table 5).

**Table 5:** Univariable analysis of prognostic factors for functional success and survival after biliary drainage

	**Functional success**	**Survival**
**Characteristics**	**OR (95% CI)**	**HR (95% CI)**
WHO PS 2–3 vs. WHO PS 0–1	0.77 (0.19–3.19)	**3.27 (1.43–7.44)**
Presence of ascites	0.48 (0.12–1.93)	**2.36 (1.14–4.91)**
Primary tumor removed	1.92 (0.38–9.65)	**0.26 (0.11–0.63)**
Hemi-hepatectomy or segmental resection	2.60 (0.65–10.38)	0.71 (0.36–1.42)
More than 1 previous systemic therapy lines	0.40 (0.10–1.54)	1.88 (0.95–3.72)
IH vs. EH cause of obstruction	1.76 (0.43–7.19)	0.82 (0.40–1.68)
Infrahilar vs. hilar/suprahilar obstruction	0.56 (0.12–2.54)	1.19 (0.55–2.61)
≥ 5 IH metastases	0.37 (0.09–1.52)	**3.18 (1.45–6.98)**
Overall estimated hepatic invasion of > 50%	0.50 (0.04–6.28)	**5.30 (1.29–21.75)**
Pre-procedural bilirubin^a^	0.25 (0.06–1.00)	**2.23 (1.13–4.59)**
Pre-procedural LDH^a^	0.25 (0.06–1.06)	**2.78 (1.30–5.94)**
Technical success of biliary drainage	-	**0.33 (0.13–0.84)**
Functional success of biliary drainage	-	**0.19 (0.08–0.44)**^**b**^
Biochemical success of biliary drainage	-	**0.16 (0.06–0.39)**
Administration of subsequent treatment	-	**0.11 (0.04–0.33)**

*EH* Extrahepatic, *HR* Hazard Ratio, *IH* Intrahepatic, *LDH* Lactate dehydrogenase, *OR* Odds Ratio, *WHO PS* World Health Organization Performance Status

^a^For laboratory measures, we compared values above with values below the median

^b^This HR was adjusted for WHO PS (2–3 vs. 0–1). The unadjusted HR was 0.22 (95% CI 0.09–0.50)

## Discussion

Allowing oncologic therapy, treating cholangitis, or relieving symptoms of jaundice are major goals of biliary drainage in the palliative treatment of mCRC patients with malignant biliary obstruction. This study describes 37 mCRC patients with malignant biliary obstruction who were treated with biliary drainage, of which half of the patients achieved the intended goal of biliary drainage. When biliary drainage was performed with the intention to allow further oncologic therapy, functional success was achieved in 12 (46%) patients. This is in line with percentages of 40% [9] and 56% [7] reported previously. In the series of Nichols et al., only 6 of 36 (17%) patients treated with biliary drainage received subsequent systemic therapy, but patients who might have been ineligible for systematic therapy at baseline were also included [4].

When biliary drainage was performed to relieve symptoms, 4 (50%) patients reported improvement of symptoms. Other studies reported that biliary drainage could indeed provide considerable improvement of jaundice-related symptoms in patients without any plans to initiate further oncologic therapy [8, 14, 15]. Others, however, stressed the morbidity and short-term mortality after biliary drainage hampering the improvement of the overall quality of life [6, 9, 16–18].

Our cumulative technical success rate of 84% is in line with previous studies reporting technically successful biliary drainage in 63 to 100% of mCRC patients [7, 9, 11]. Yet, functional success was only achieved in 50% and biochemical success in 44% of patients. This could be explained by the possible presence of additional obstructed segments, along the ones opacified and technically successfully treated, that remained unopacified during cholangiography and were left untreated and undrained. Especially, hilar location and complex strictures have been reported to be associated with insufficient decrease of bilirubin, because they are more difficult to reach and challenging to drain effectively [10, 19, 20]. Also, the presence of liver metastases was associated with a lower rate of effective drainage [19, 21], and serum bilirubin level rarely normalized in patients with extensive liver metastases, because of mechanical biliary obstruction and parenchymal replacement [22].

Advanced palliative systemic therapy regimens showed prolonged overall survival in mCRC patients [23, 24], but some agents are contraindicated in patients with malignant biliary obstruction [23–25]. Biliary drainage aims to allow such regimens. We observed significantly longer survival in patients who received further oncologic therapy, regardless of baseline WHO performance status. Similarly, improved survival from 1-2 months after biliary drainage without subsequent systemic therapy to 9-12 months in those receiving subsequent systemic therapy has been reported in the literature [4, 7–9].

Prognostic factors of favorable outcome after biliary drainage, such as certain baseline or radiological findings, might improve patient selection and could be used to develop a risk prediction model to determine who is most likely to benefit from biliary drainage. Previous studies found that baseline characteristics, such as absence of fever and ascites, previous liver surgery, and performance status are associated with successful biliary drainage [7]. Also, hilar bile duct stricture and large hepatic tumor load were suggested as prognostic factors for unsuccessful drainage (in terms of bilirubin reduction) [7, 10, 11], but this was not confirmed in our study. For survival, however, we identified several risk factors of improved survival.

### Strengths and Limitations

We only included mCRC patients and reported outcomes by indication of biliary drainage to describe clinical outcomes in a homogenous population. The outcome ‘functional success’ can be used to evaluate biliary drainage for different indications. Especially in palliative settings, outcome definition tailored to intended goals of treatment is more indicative to assess effects of treatment. Radiological characteristics were collected prospectively and were independently analyzed by two radiologists in training who were blinded to outcomes.

This study has several limitations. Clinical data were retrospectively collected from electronic medical records. In some patients, it was difficult to determine the intended goal of biliary drainage retrospectively. Objective and quantitative measures for symptom relief were not available. Patients deemed ineligible for biliary drainage with reasons to refrain from biliary drainage were not included. All patients were treated in a single tertiary center, which could hamper generalizability to other mCRC populations. The small sample size did not allow subgroup analysis and most associations could not be adjusted for confounding factors. While the association between functional success and survival was adjusted for baseline performance status, it might be confounded by other characteristics. Quality of life could not be measured retrospectively. The study included patients treated during a 9-year period and improvement of procedural techniques and systemic treatments could have influenced outcomes.

### Implications for Clinical Practice and Future Research

Biliary drainage can be offered to mCRC patients to allow subsequent oncologic therapy or provide symptom relief. It could provide significant survival benefit if functional success is achieved, especially in whom biliary drainage allows administration of further oncologic therapy. The clinical significance of the survival benefit of 31 days found in patients successfully treated with biliary drainage to relieve jaundice-related symptoms will depend on the occurrence of adverse events and the duration and extent in which symptoms remain alleviated. Treatment decisions should be made in a multidisciplinary hepatobiliary team, discussing patient and disease characteristics, radiographical findings, drainage options, and possibilities of further oncologic therapy. Rates of functional success and survival benefit, as well as the potential necessity of multiple interventions and the risk of adverse events should be taken into account when counseling the patient. Outcomes of biliary drainage could not be compared with the outcomes of abstaining from biliary drainage, because the latter were not included in this study. Large studies with mCRC patients are needed to identify prognostic factors for functional success and survival after biliary drainage. Prediction models to predict outcomes after biliary drainage might help guide in selecting patients who benefit most from it. This is especially relevant for mCRC patients that are otherwise eligible for further oncologic treatment, since survival can significantly improve with palliative systemic therapy regimens [23, 24]. Symptom relief and quality of life, major goals of palliation, should also be assessed.

## Conclusion

This study demonstrated that functionally successful biliary drainage is achieved in half of mCRC patients with malignant biliary obstruction. Multiple interventions and different modalities are frequently needed, and adverse events are reported in nearly half of the patients. This should be taken into account during shared decision-making with the patient and during multidisciplinary hepatobiliary meetings. Significantly longer survival was observed in patients in whom biliary drainage allowed subsequent oncologic therapy.
